# Association of NQO2 With UDP-Glucuronosyltransferases Reduces Menadione Toxicity in Neuroblastoma Cells

**DOI:** 10.3389/fphar.2021.660641

**Published:** 2021-05-10

**Authors:** Monivan Chhour, Pierre Perio, Regis Gayon, Hélène Ternet-Fontebasso, Gilles Ferry, Françoise Nepveu, Jean A. Boutin, Jan Sudor, Karine Reybier

**Affiliations:** ^1^ Pharma-Dev UMR 152, Université de Toulouse, IRD, UPS, Toulouse, France; ^2^ Flash Therapeutics, Parc Technologique du Canal, Toulouse, France; ^3^ Biotechnologie, Pharmacologie Moléculaire et Cellulaire, Institut de Recherches Servier, Croissy-sur-Seine, France

**Keywords:** NQO2, UGT1A6, conjugation, glucuronide, menadiol, cellular toxicity

## Abstract

The balance between detoxification and toxicity is linked to enzymes of the drug metabolism Phase I (cytochrome P450 or oxidoreductases) and phase II conjugating enzymes (such as the UGTs). After the reduction of quinones, the product of the reaction, the quinols—if not conjugated—re-oxidizes spontaneously to form the substrate quinone with the concomitant production of the toxic reactive oxygen species (ROS). Herein, we documented the modulation of the toxicity of the quinone menadione on a genetically modified neuroblastoma model cell line that expresses both the quinone oxidoreductase 2 (NQO2, E.C. 1.10.5.1) alone or together with the conjugation enzyme UDP-glucuronosyltransferase (UGT1A6, E.C. 2.4.1.17), one of the two UGT isoenzymes capable to conjugate menadione. As previously shown, NQO2 enzymatic activity is concomitant to massive ROS production, as previously shown. The quantification of ROS produced by the menadione metabolism was probed by electron-paramagnetic resonance (EPR) on cell homogenates, while the production of superoxide was measured by liquid chromatography coupled to mass spectrometry (LC-MS) on intact cells. In addition, the dysregulation of the redox homeostasis upon the cell exposure to menadione was studied by fluorescence measurements. Both EPR and LCMS studies confirmed a significant increase in the ROS production in the NQO2 overexpressing cells due to the fast reduction of quinone into quinol that can re-oxidize to form superoxide radicals. However, the effect of NQO2 inhibition was drastically different between cells overexpressing only NQO2 vs. both NQO2 and UGT. Whereas NQO2 inhibition decreases the amount of superoxide in the first case by decreasing the amount of quinol formed, it increased the toxicity of menadione in the cells co-expressing both enzymes. Moreover, for the cells co-expressing QR2 and UGT the homeostasis dysregulation was lower in presence of menadione than for the its counterpart expressing only QR2. Those results confirmed that the cooperation of the two enzymes plays a fundamental role during the cells’ detoxification process. The fluorescence measurements of the variation of redox homeostasis of each cell line and the detection of a glucuronide form of menadiol in the cells co-expressing NQO2 and UGT1A6 enzymes further confirmed our findings.

## Introduction

Quinones represent a ubiquitous class of compounds commonly found in natural products and endogenous biochemicals or generated through re-oxidation of hydroquinones and/or catechols. They can undergo enzymatic reduction by one-electron-reductases and non-enzymatic redox cycling leading to the formation of semiquinone and deleterious reactive oxygen species (ROS) (Monks, et al., 1992; Bolton et al., 2000; O'Brien, 1991).

Quinone reductases such as NAD(P)H: quinone oxidoreductase 1 (NQO1) and NRH: quinone oxidoreductase 2 (NQO2) are two-electron reductases responsible for the detoxification of quinones due to their ability to form hydroquinones and to prevent their one-electron reduction leading to the formation of reactive semiquinone and ROS (Kappus and Sies, 1981; Bolton et al., 2000). However, in many cases, the hydroquinone (quinol) formed is not stable enough and immediately re-oxidizes to semiquinone, also inducing the concomitant production of ROS (Reybier et al., 2011) by electron transfer to oxygen. Indeed, previous studies carried out on cells have shown that the metabolism of some quinones (ortho or para) by quinone reductase 1 and 2 was responsible for a significant production of free radicals (Cassagnes et al., 2015). The properties of the hydroquinone determine whether QR functions as a protective antioxidant or a pro-oxidant activator (Klaassen et al., 2013) for example for the bioactivation of chemotherapeutic quinones (Walton et al., 1991; Parkinson and Hergenrother, 2015; Zhang et al., 2018).

**SCHEME 1:**
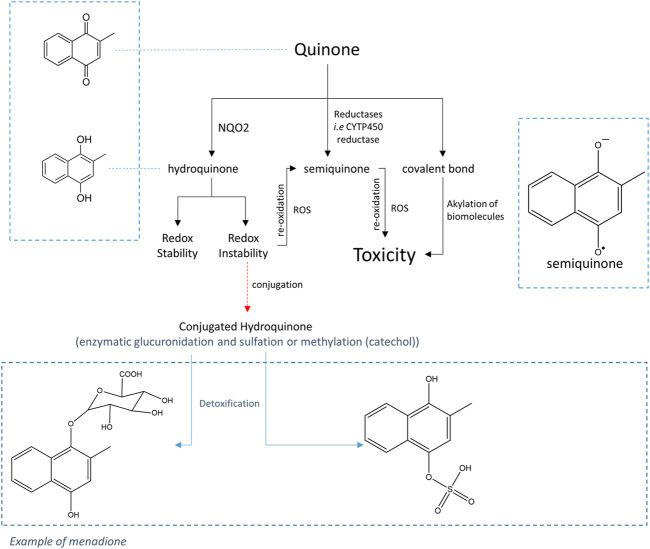
General pathway for the metabolism of quinones.

Moreover, analyses carried out on different types of cells have shown that the toxicity of quinones depends on the nature of the quinone but also on the nature of the cells. Thus, in the case of CHO cells, two contradictory characteristics of NQO2 have been highlighted (Cassagnes et al., 2015); while the presence of NQO2 decreases the toxicity of menadione, it worsens that of adrenochrome, an ortho-quinone. Moreover, studies carried out with K562 cells which naturally express NQO2, or with neuronal cells (SH-SY5Y or primary neurons) modified to overexpress NQO2, have shown that for these cells, the presence of NQO2 was responsible for the increased toxicity for o- and p-quinones (menadione and adrenochrome, respectively) (Cassagnes et al., 2018). The hypothesis that has been proposed is that cooperation with a conjugating enzyme which is able to react with the unstable reduced form (quinol) thus preventing its re-oxidation, is necessary to effectively detoxify the quinones (Bock et al., 1987), as proposed by Nishiyama et al. for QR1 (Nishiyama et al., 2010). Thus, in this case, the detoxifying character of NQO2 towards toxic quinones would depend on the expression of conjugating enzymes in the oxydo-reductase expressing cells as depicted in Scheme 1.

The main conjugation reactions involving hydroquinones are sulfation, glucuronidation and methylation reactions catalyzed by sulfotransferases (SULT) (Chan and O'Brien, 2003), UDP-glucuronosyltransferases (UGT) and catechol-O-methyltransferases (COMT), respectively (Taskinen et al., 2003; Shangari et al., 2005). Experiments on animal models (rat and rabbit) treated with menadione have demonstrated the presence of menadiol-glucuronide in bile and urine (Richert, 1951; Losito et al., 1967; Thompson et al., 1972). In the current state of knowledge, the family of human UGTs includes 16 isoforms excluding UGT allozymes. According to Nishiyama et al. (2008), only two UGT enzymes, UGT1A6 and UGT1A10, are capable to react with the reduced form of menadione, UGT1A6 being ten times more effective than UGT1A10 in the conjugation reaction to produce the stable, readily eliminable glucuronides.

To test this hypothesis, a specific line of neuroblastoma cells genetically modified to express both NQO2 and the specific conjugation enzyme UGT1A6 was produced to investigate the effect of the co-expression of both enzymes on menadione toxicity. In this paper, different techniques were applied including LC-MS (liquid chromatography-coupled to mass spectrometry) for the quantification and specific identification of ROS produced as a result of quinone metabolism and for the detection of the conjugated form of the hydroquinone. Additionally, fluorescence assays were used to better understand the dysregulation of redox homeostasis under quinone treatment.

## Material and Methods

### Chemicals

Menadione, phosphate buffer (DPBS), dihydroethidium (DHE), thiazole orange (TO), diethylene triamine penta-acetic acid (DTPA), uridine 5'-diphospho-glucuronic acid (UDPGA) and potassium nitrosodisulfonate (NDS) were purchased from Sigma-Aldrich (Saint Quentin Fallavier, France). 1-benzyl-1,4-dihydronicotinamide (BNAH) was purchased from TCI Chemicals (Zwijndrecht, Belgique) and dimethylsulfoxyde (DMSO), FBS (Foetal Bovin serum), DMEM/F12, ammonium acetate, methanol, acetonitrile from ThermoFisher Scientific (Illkirch, France), DMPO (5,5-dimethyl-1-pyrroline N-oxide) from Dojindo (Kumamoto, Japon) and Trypsine from Corning (Boulogne-Billancourt, France). S29434 (N-[2-(2-methoxy-6H-dipyrido[2,3-a:3,2-e] pyrrolizin-11-yl)ethyl]-2-furamide) was provided by the Institut de Recherches Servier (Croissy-sur-Seine, France). 2-Hydroxyethidium (2OH-E+) was synthesized by the reaction of hydroethidine (DHE) with potassium nitrosodisulfonate (NDS) in the proportion 2/1 (Zielonka et al., 2008). The synthesis was performed by mixing: 200 µl of hydroethidine (20 mM) in 4 ml of phosphate (50 mM) DPTA buffer (100 µM), 24 ml of water, and 8 ml of NDS (1 mM) with gentle agitation. The mixture was kept at room temperature for 2 h, then purified by solid phase chromatography (SPE).

### Cell Culture and Double Transfected Cell Production

Native SH-SY5Y (ATCC® CRL-2266) and transduced cells were cultivated in T75 CellBIND® flask (Corning, NY, United States) using EMEM/Ham’s F12 (1:1) media (Corning, NY, United States) complemented with 10% FBS (Biowest, Nuaillé, France), 2 mM L-Gln (Corning, NY, United States) and 1% Penicillin/Streptomycin (Corning, NY, United States), seeded at 66,500 cells/cm^2^ and incubated at 37°C/5% CO_2_. The UGT1A6 or hNQO2 overexpressing cell lines were generated by transducing native cells at MOI40 in presence of 2 mg/ml Polybrene (Sigma, Saint Louis, MO, United Sates) by using integrative lentivectors (iLV) expressing EF1-UGT1A6 or EF1-hNQO2 both driven by the human EF1a promoter, custom produced by Flash Therapeutics (LentiSTART batches, Toulouse, France). One week later, the hNQO2 overexpressing cell line was transduced in the same conditions by using iLV-EF1a-UGT1A6 to obtain the co-expressing hNQO2 and UGT1A6 cell line.

### Western Blotting

Proteins were extracted using the RIPA buffer (Sigma, Saint Louis, MO, United States). Protein concentration was assayed using Pierce BCA Protein Assay Kit (Thermo Fisher, Courtaboeuf, France). Total proteins (5 µg) were loaded onto an SDS-PAGE gel with a gradient of 4%–12% and separated for 50 min at 200 V. The separated proteins were blotted onto a PVDF membrane for 50 min at 20 V. Western blots were probed with either an anti-UGT1A6 antibody (ab157476, diluted 1:1000, Abcam, Cambridge, UK), an anti-QR2 (a.k.a. NQO2) antibody (H00004835-A01, diluted1:500, Abnova, Taipei City, Taiwan) or an anti-Actin antibody (ab8224, diluted 1:2000, Abcam, Cambridge, United Kingdom) as a loading control. ECL signals were detected with the Pierce Fast Western Blot Kit (Pierce Biotechnology, Rockford, IL, United States) and revealed by autoradiography.

### EPR Analysis

Electron paramagnetic resonance (EPR) spectra were recorded at room temperature using a Bruker EMX-8/2.7 EPR spectrometer (9.86 GHz) equipped with a high-sensitivity cavity (4119/HS 0205) and a Gaussmeter (Wissembourg, France). A flat cell (FZKI160−5 × 0.3 mm, Magnettech, Berlin, Germany) was used for the analysis. The EPR analysis were performed using the following parameters: magnetic field: 3460–3560 G; sweep: 100 G; sweep time 83.89 s; time constant: 81.92 ms; number of scans: 1; amplitude modulation: 1 G; frequency modulation: 100 kHz; microwave power: 20 mW; attenuation: 10 dB; gain 2.105. The intensity of the spectra was calculated by double integration of the signal using the WINEPR software. EPR were carried out on cells homogenates. Cell homogenates were prepared from 5 million of neuroblastoma cells using a homogenizer (Dounce Kimble tissue grinder). The lysates were recovered and centrifuged at 20,000 × g for 30 min at 4°C before treatments and analysis. The detection of superoxide was carried out using DMPO as spin trap. The final concentrations used were: DMPO 50 mM, BNAH 100 μM, UDPGA 2 mM, menadione 100 μM. When needed, the cells were incubated for 30 min at 37°C with the specific NQO2 inhibitor S29434 at 20 μM in an oscillating shaker before performing the EPR assays.

### LC-MS Analysis

**FIGURE 1:**
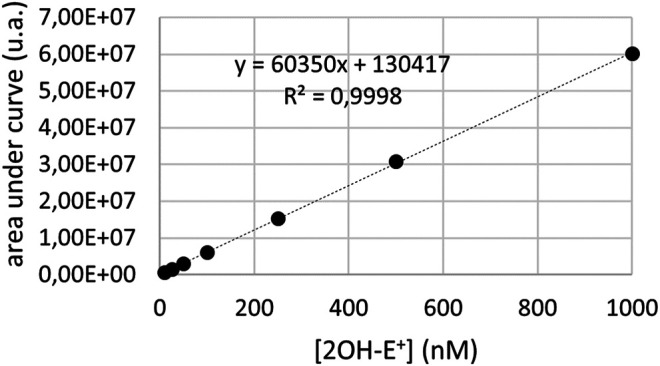
LC-MS calibration curve for the quantification of 2OH-E+. Such calibration was obtained from analyzing known quantities of pure compound with the same system, to quantify superoxide radicals.

Superoxide radicals were analyzed by liquid chromatography coupled with mass spectrometry (LC-MS) as previously described (Zielonka et al., 2008; Tsamesidis et al., 2020a). Ultimate 3000 UHPLC system consisting of a solvent organizer SRD-3600 with degasser, a high pressure binary gradient pump HPG-3400RS, a thermostated autosampler WPS3000TRS, an oven TCC3000SD and an UV-Visible detector DAD3000 (ThermoFisher Scientific, Courtaboeuf, France) and LTQ-Orbitrap XL ETD mass spectrometer (ThermoFisher Scientific, Courtaboeuf, France), was used. Xcalibur software was used for raw data acquisition and results analyses. The detection of superoxide radicals was performed with dihydroethidium (DHE) *via* the detection of 2OH-E+ using a positive electrospray ionization (ESI). Quantitative analysis was performed using Xcalibur software by integrating the signal obtained for the corresponding extracted mass (m/z 330 for 2OH-E+) chromatograms. In order to confirm the identity of the detected compounds, the mass spectrometer was used in FTMS mode at a resolution 15,000 for 2OH-E+. The chromatographic separation was achieved on a Kinetex EVO C18 column (2.1 × 100 mm, 1.7 μm particle size) (Phenomenex, Le Pecq, France) at a flow rate of 400 μl/min and column temperature set at 50°C using an aqueous mobile phase containing acetonitrile (Tsamesidis et al., 2020a). The cells were resuspended in the DPBS at a density of 5 million cells. Reagents were added in the following order: BNAH (25 µM), UDPGA (2 mM), DHE (10 µM) and menadione (25 µM). The cells were then incubated in the dark under agitation at 37°C for 30 min. The cells were then centrifuged at 120 g. The pellet was recovered and washed with ice-cold DPBS solution. The cells were then lysed using 100 µl Triton X-100 solution (1% in DPBS). The solution was then half diluted with methanol/ammonium acetate solution (50/50) and centrifuged at 20,000 g for 30 min. The supernatant was recovered for analysis. Intracellular concentrations of superoxide were deduced from the calibration curve. Calibration curves (Figure 1) were obtained by preparing series of dilutions of 2-hydroxyethidum at 1000, 500, 250, 100, 50, 25 and 10 nM in the solvent methanol/ammonium acetate (25/75), for a final volume of 200 μl, and determining peak areas by UHPLC-MS. Glucuronide identification was performed with the previously described UHPLC-MS system using a MS/MS instrument method to attest the presence of the glucuronide moiety. The separation was done on a Kinetex Biphenyl column (2.1 × 100 mm, 1.7 μm particle size) (Phenomenex, Le Pecq, France) at a flow rate of 0.5 ml/min and a column temperature set at 40 °C using a linear gradient of eluant A, H2O + 0.1% formic acid, and eluant B, ACN + 0.1% formic acid. An APCI ionization source was used in negative mode and 2 independent scan events was used. The first scan event corresponds to a full MS analysis in the m/z range 115–800 in FTMS mode at a resolution of 15,000. The second scan event, performed for MS/MS structural confirmation analysis, was focused on the mass/charge ratio of the glucuronide ion (m/z 349) in FTMS mode at a resolution of 7,500. The Collision Induced Dissociation (CID) fragmentation was set at a normalized collision energy of 35 (arbitrary units).

### Thiazole Orange Experiments

The test used derived from the "LUCS" (Light-Up Cell System) technology developed by AOP (Antioxidant Power) company (Derick et al., 2017; Gironde et al., 2020). Neuroblastoma cells were seeded onto the 96-well plate (Corning) at 35,000 cells per well. The plate was incubated for 24 h at 37°C with 5% CO_2_. The medium was then removed and 25 μl of thiazole orange 2 μM were added. After 60 min of equilibration, the cells were treated by 25 µl of menadione (6–50 µM), 50 µl of BNAH (25 µM), 50 µl of UDPGA (2 mM) and thiazole orange 2 µM was added to complete the final volume to 100 μl. The final DMSO concentration was 4%. The fluorescence of the TO was measured using a Xenius® fluorimeter from SAFAS (Monaco, driven by SP2000 software version 7.4.13.0). Analyses were performed every 2 min for 240 min (λex: 505 nm/λem: 535 nm) 10 nm bandwidth with full filtering. The photomultiplier voltage (PM) was set to 600 V.

For all graphes, the errors bars correspond to standard deviations. The experiments have been performed at least 3 times. The results were compared using the Student's *t*-Test with ns *p* > 0.05, *p < 0.05, **p < 0.01 and ***p < 0.005.

## Results and Discussion

To evaluate the impact of the association of NQO2 with UGT1A6, different methods were applied such as the measurement of the production of ROS by LC-MS and fluorescent assays or the modification of the cell redox homeostasis when cells were treated with menadione.

### Building of the Double Transfected Cells

**FIGURE 2:**
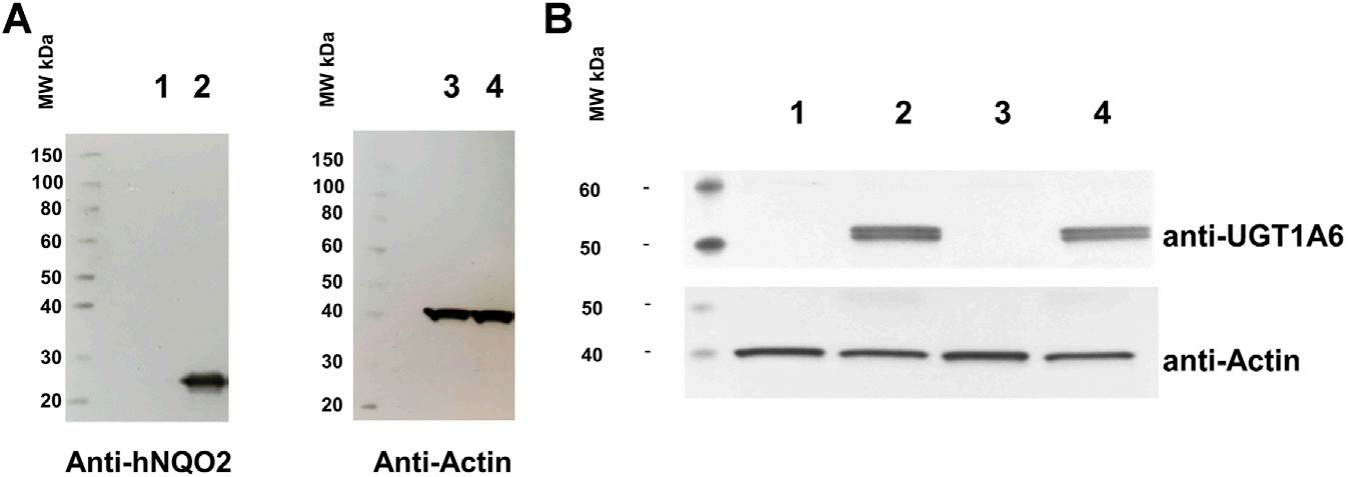
Analyses of the double stable expression of UGT and NQO2 in SH-SY5Y cells. **(A)** Western blot analysis of the original SH-SY5Y cell line stably expressing NQO2. Lane 1 and 3: SH-SY5Y; Lane 2 and 4: SH-SY5Y expressing both UGT1A6 and NQO2. **(B)** Western blot of the double stable SH-SY5Y expressing UGT1A6. Lane 1: SH-SY5Y naive cells; lane 2: SH-SY5Y UGT1A6; lane 3: SH-SY5Y NQO2; lane 4: SH-SY5Y stably containing both NQO2 and UGT1A6.

Cells expressing stably the NQO2 and the UGT1A6 genes were obtained by transduction of cells using classical, virus transduction techniques integrative lentiviral vectors. The NQO2-only expressing construct has been described previously (Cassagnes et al., 2015). The evidence for the expression of NQO2 in this cell line is presented again in Figure 2A. Double stable cells expressing both genes were obtained and characterized with WB expressions of both protein (Figure 2B). Furthermore, we checked that the naïve cells SH-SY5Y did not express naturally a UGT protein (lane 1) nor that the expression of NQO2 did not induce the expression of NQO2 in the parent cells (lane 3).

### Effect of UGT1A6 on the Production of ROS

**FIGURE 3:**
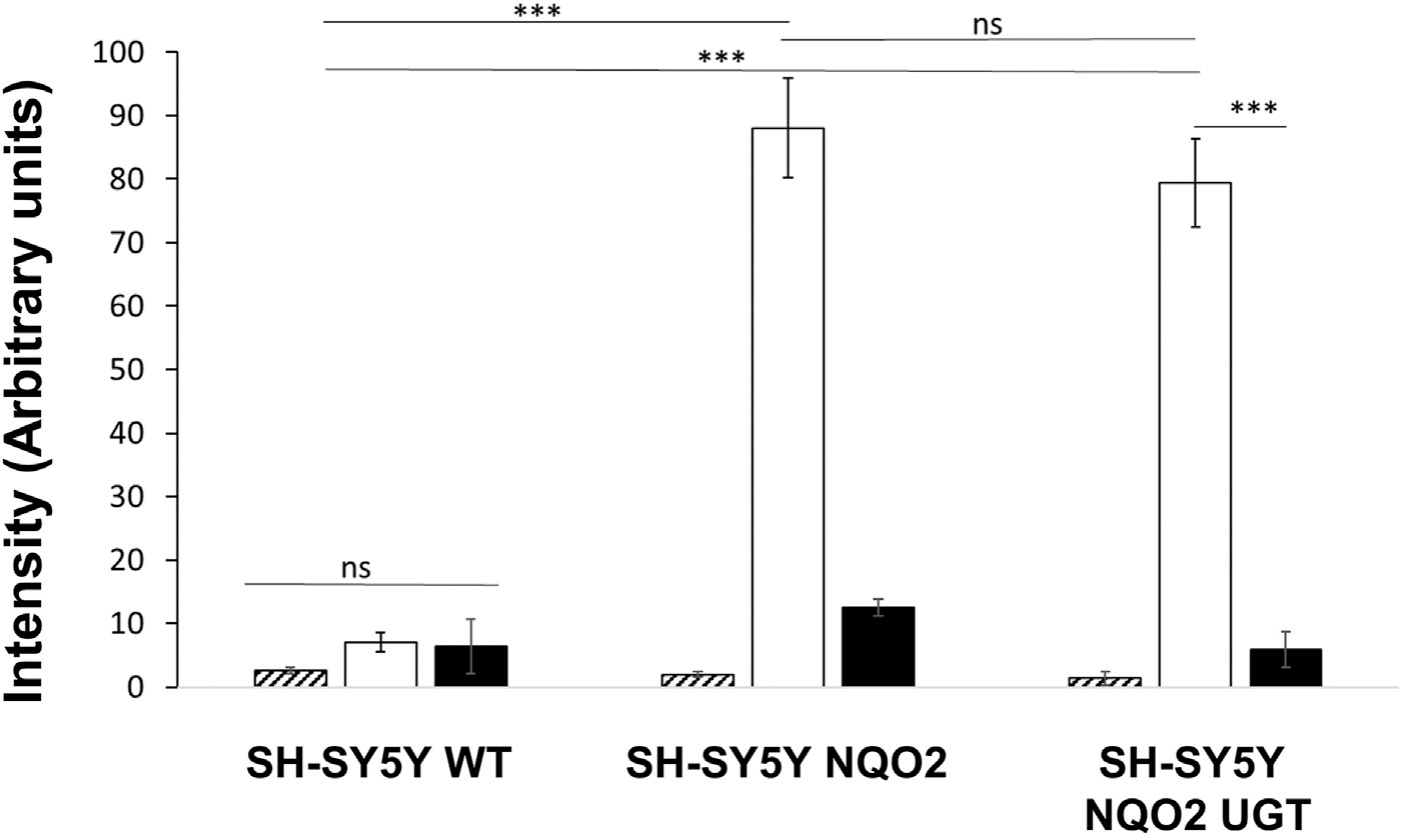
EPR measurement of reactive oxygen species produced by the cells. ROS produced by the 3 cell lines, SH-SY5Y-WT, SH-SY5-NQO2 and SH-SY5Y-NQO2-UGT were analyzed by EPR after 2 min incubation with menadione (100 µM), BNAH (100 µM), UDPGA (2 mM), DMPO (50 mM) and pre-incubated 30 min with S29434 (20 µM) when needed. Hatched histograms: menadione, no co-substrate BNAH; clear histograms: menadione and BNAH; dark histograms: menadione, BNAH and 29434. The results correspond to 3 independent experiments. ns *p* > 0.05, ***p ≤ 0.005.

ROS production derived from the metabolism of menadione was first analyzed by EPR for the three cell lines: the wild-type cells (SH-SY5Y-WT), the cells overexpressing only NQO2 (SH-SY5Y-NQO2), and both NQO2 and UGT1A6 (SH-SY5Y-NQO2-UGT1A6). Since the spin adduct formed after DMPO radical trapping is rapidly metabolized into the cell (Samuni et al., 1986), all the experiments were conducted on cells homogenates. The cell homogenate was prepared from 5 million neuroblastoma cells using a homogenizer (Dounce Kimble tissue grinder). The spectra were recorded after 2 min of incubation with BNAH (100 μM) (the synthetic co-substrate of NQO2), UDPGA (2 mM) (UGT co-substrate) and menadione (100 μM). The corresponding production of ROS, evaluated by the double integration of the signal, is presented in Figure 3. In contrast to our previous results obtained by EPR on intact cells (Cassagnes et al., 2015), the intensity recorded on the wild-type cells homogenates was very weak meaning that the contribution of one-electron reductases is minimal, if any. This result is probably due to the use of cell homogenates that significantly dilutes the intracellular NAD(P)H concentration, essential to the enzymatic machinery of most reductases as NADPH-cytochrome P-450 reductase (Giulivi and Cadenas, 1994). On the contrary, as we previously reported (Cassagnes et al., 2018), in the presence of NQO2, a remarkable increase of ROS production was observed (40-fold) compared to the parent cell expressing low levels of NQO2. This increase was due to NQO2 and the re-oxidation of menadiol under enzymatic reduction, as no significant increase was observed in the absence of the co-substrate of NQO2, BNAH, or when the enzyme was inhibited by its specific inhibitor S29434 (Boutin et al., 2019). Working on cell homogenates has facilitated the interaction of menadione with the cytosolic enzyme NQO2 and its co-substrate giving rise to high amounts of menadiol and then to radicals. The experiments have also been done with the line SH-SY5Y-UGT. As expected, an increase in the amount radical produced was measured in presence of menadione whereas no significant difference was obtained by adding S29434, like for the WT cell line (data not shown) because this ROS production being insensitive to the NQO2 inhibition is mostly due to alternate enzymatic processes such NQO1 or Cyt P450. However, no significant difference was observed on the same cells overexpressing the conjugating enzyme, UGT1A6. It can be assumed that under these conditions, the menadiol re-oxidation rate is higher than that of the conjugation one, which in turn would lead to the overproduction of the reduced form when the detoxification process is overwhelmed.

**FIGURE 4:**
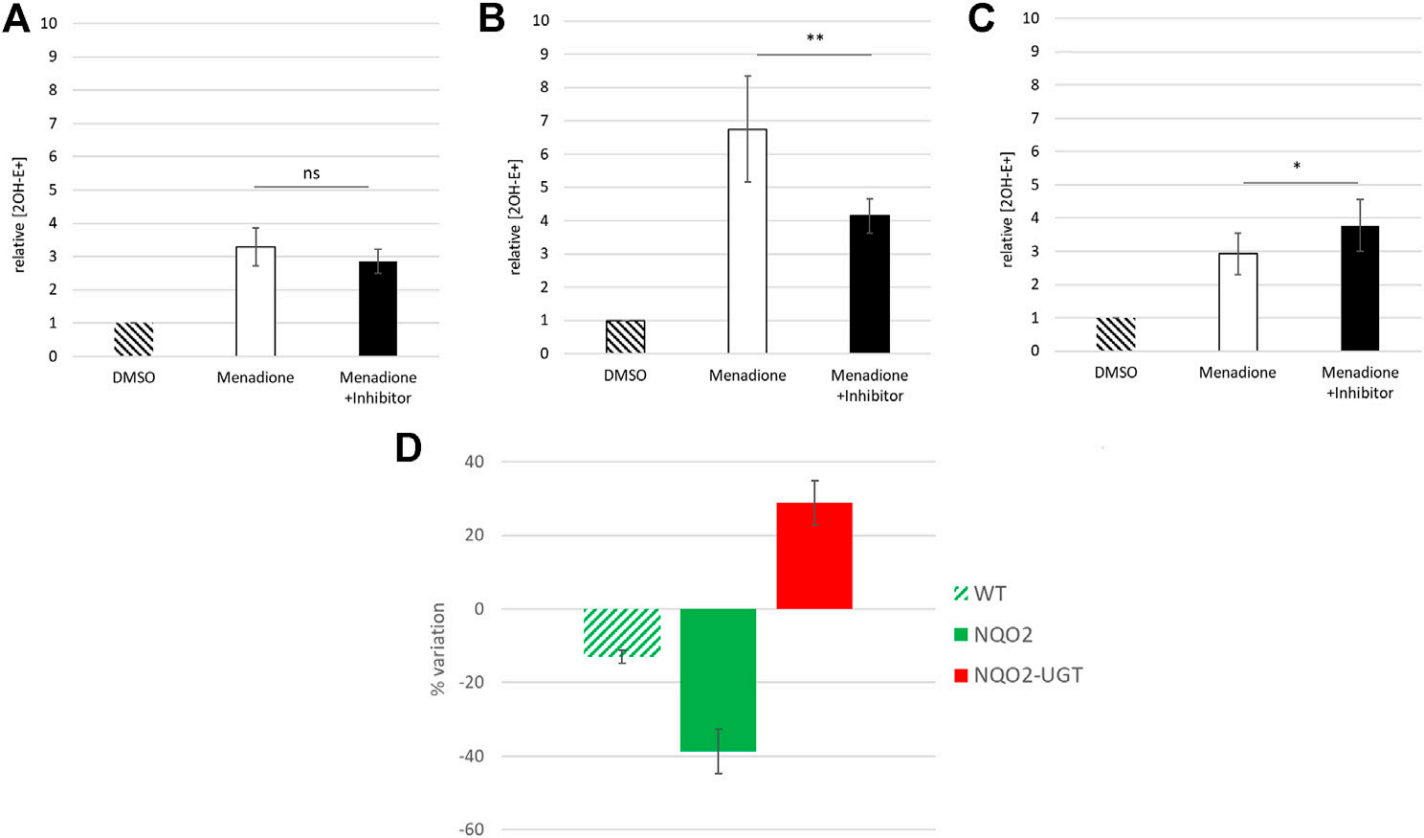
Relative intracellular concentration of superoxide radicals measured by LC-MS. Relative concentration of superoxide radicals produced after 30 min incubation with menadione (25 µM) and BNAH (25 µM) by SH-SY5Y-WT **(A)** SH-SY5-NQO2 **(B)** and SH-SY5Y-NQO2-UGT in presence of UDPGA (2 mM) **(C)** compared to DMSO. When needed the cells were pre-incubated 30 min with the inhibitor S29434 (20 µM). **(D)** represents the percentage of variation of the production of superoxide on the three cell lines when NQO2 is inhibited (production increase in red, production decrease in green). The results correspond to 3 independent experiments. ns *p* > 0.05, *p ≤ 0.05, **p ≤ 0.01.

We thus moved to intact cells, in which the detection of superoxide, the primary radical formed under the menadione metabolism, was measured by LC-MS for the 3 cell lines. These sets of experiments were based on the use of dihydroethidium (DHE) which gives a specific adduct, the 2-hydroxyethidium (2OH-E^+^) after reaction with superoxide (Zielonka et al., 2008; Kalyanaraman et al., 2017; Tsamesidis et al., 2020a; Tsamesidis et al., 2020b) that can be detected by MS (m/z 330). The three SH-SY5Y lines were incubated for 30 min in the presence of BNAH (25 μM), UDPGA (2 mM), DHE (10 μM) and menadione (25 μM). Since the incubation time is longer than for the EPR analyses, the menadione concentration was reduced from 100 μM to 25 μM to prevent cell death. The cells were then lysed, centrifuged and the supernatant was analyzed by LC-MS. The chromatographic signal corresponding to 2-hydroxyethidium was then integrated and the superoxide concentration deduced from the calibration curve obtained from the synthetic 2OH-E^+^ (Figure 1). The results obtained with each cell line are presented in Figure 4. As expected, the metabolism of menadione by one-electron-reductases increased the production of superoxide by 3.28 ± 0.57 compared to DMSO (relative concentration) (Figure 4A) in contrast to what was observed on cell homogenates and no significant decrease in superoxide production was observed by addition of the specific inhibitor with a relative concentration equal to 2.86 ± 0.36. For cells overexpressing NQO2 (Figure 4B), the results confirmed that NQO2 was responsible for an increase in the superoxide production that reached a relative concentration of 6.75 ± 1.58 compared to DMSO. In turn, this amount was decreased to 4.14 ± 0.51 in the presence of S29434, the NQO2 inhibitor. When both NQO2 and UGT were active, the relative amount of radical produced was lower (2.93 ± 0.62). More importantly, the addition of the specific NQO2 inhibitor induced a re-increase of the relative concentration to 3.77 ± 0.78 (Figure 4C). This last result demonstrates that the amount of superoxide produced and therefore the toxicity of menadione is higher when the detoxification chain is broken by inhibition of NQO2. The toxicity of menadione appears to be reduced when NQO2 is associated with UGT1A6. These results are illustrated in Figure 4D representing the ratio of the superoxide production in the presence of menadione for the 3 cell lines with or without the NQO2 inhibitor. This set of experiments for intracellular superoxide quantification demonstrates the beneficial cooperation between NQO2 and a conjugating enzyme to decrease the toxicity of menadione. Indeed, this cooperation makes it possible to block by conjugation the unstable menadiol avoiding its reoxidation and the establishment of a futile cycle (Janda et al., 2020) that is deleterious to the cell, by enhancing the amount of produced ROS.

### Effect of UGT1A6 on the Cellular Homeostasis

**FIGURE 5:**
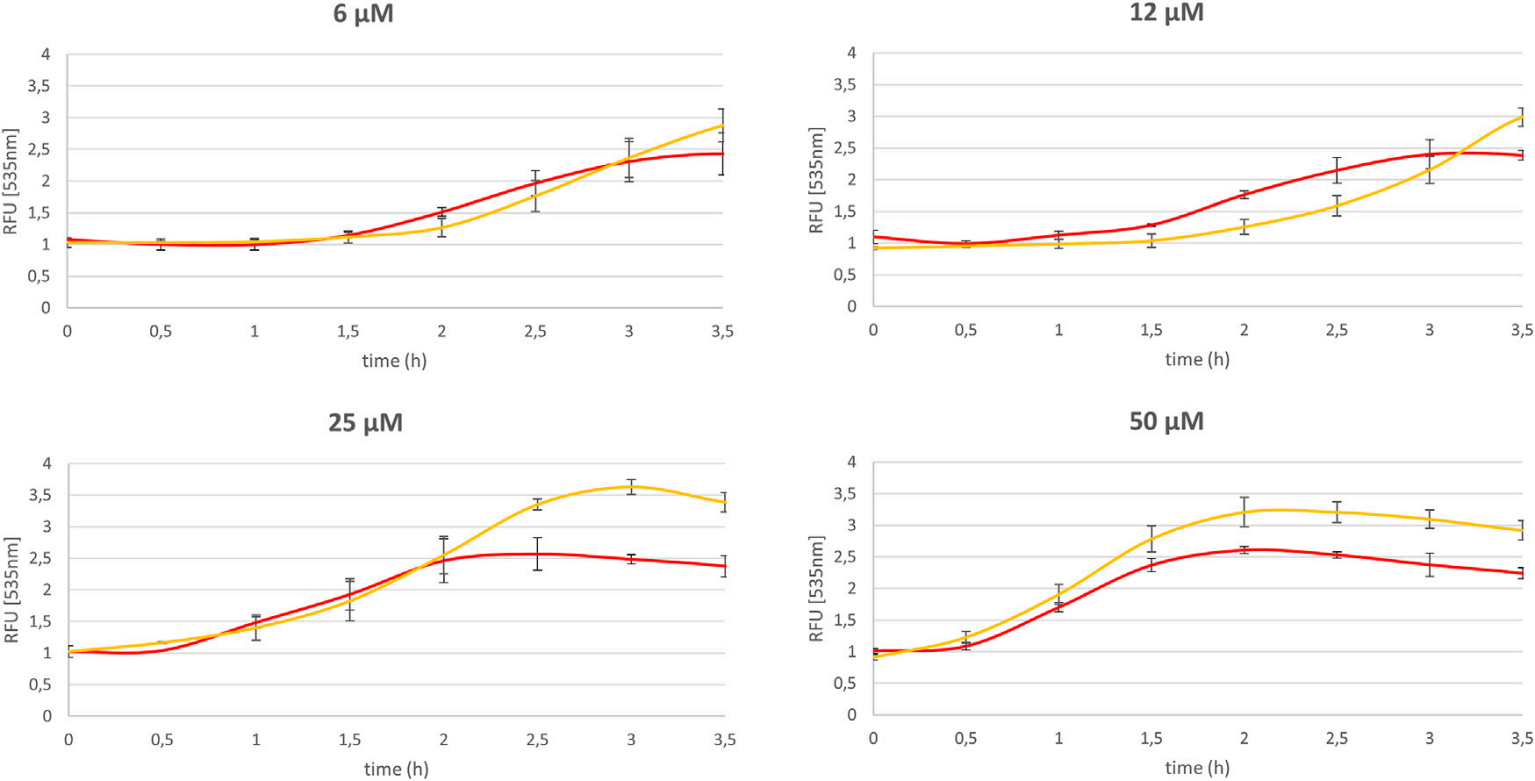
Impact of NQO2 on the menadione mediated homeostasis dysregulation. Relative thiazole orange fluorescence (2 µM, λex/em : 505/535 nm) measured for SH-SY5Y-NQO2 (yellow curve) and SH-SY5Y-NQO2 previously treated 30 min with S29434 (20 µM) (red curve) in presence of menadione (6, 12, 25 and 50 µM) and BNAH (50 µM) and compared to the one measured with DMSO. The results correspond to 3 independent experiments.

**FIGURE 6:**
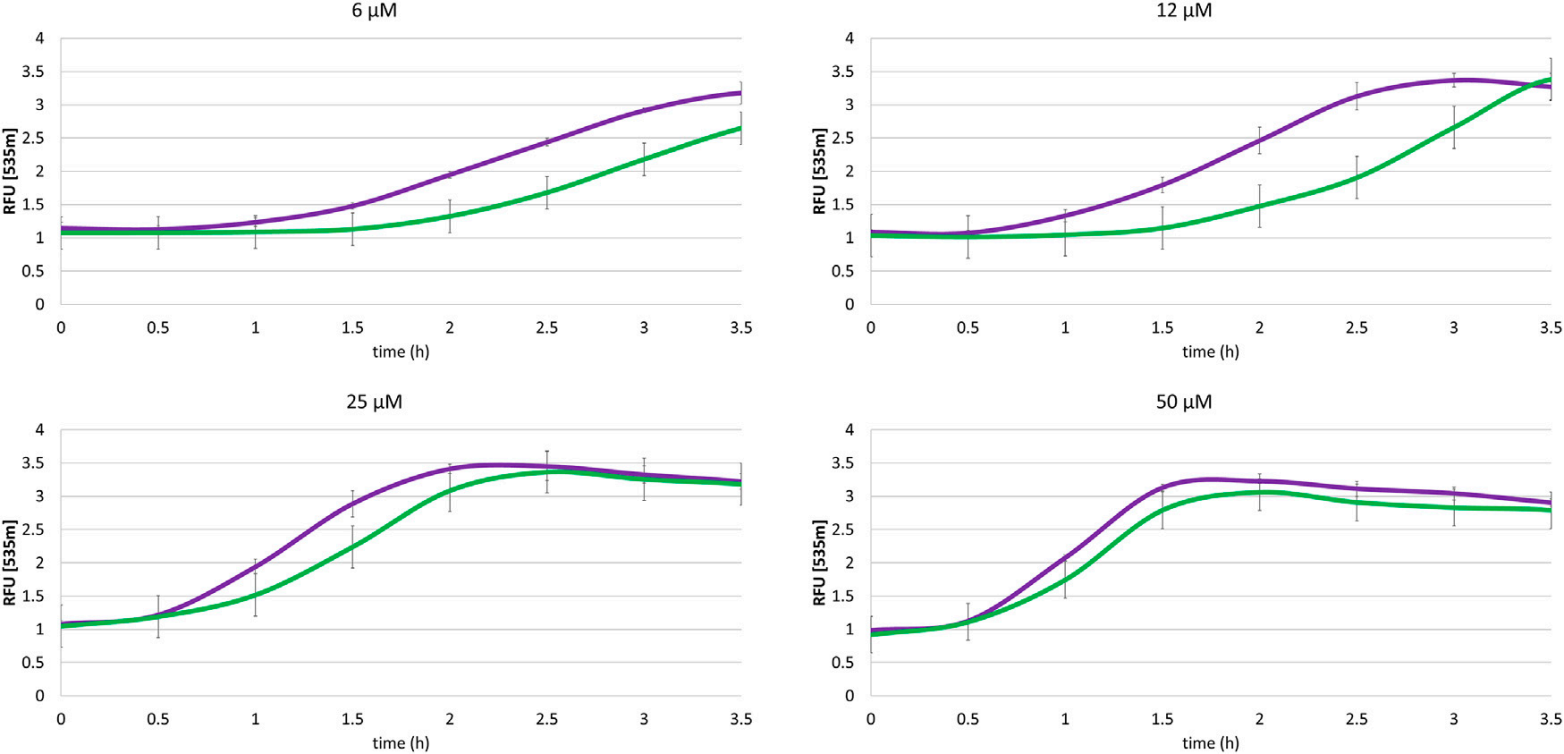
Impact of the association NQO2-UGT on the menadione-mediated cellular homeostasis dysregulation. Thiazole Orange fluorescence (2 µM, λex/em: 505/535 nm) for SH-SY5Y-NQO2-UGT (green curve) and SH-SY5Y-UGT previously treated 30 min with S29434 (20 µM) (purple curve) measured in presence of menadione (6, 12, 25 and 50 µM), BNAH (50 µM) and UDPGA (2 mM). The results correspond to 3 independent experiments.

We demonstrated that the cellular toxicity of quinones is diminished by the co-expression of a conjugating enzyme which decreases the production of ROS and especially of superoxide, by stabilizing the end-product of the reduction step. To confirm this finding, the dysregulation of the redox homeostasis of each cell type under metabolism of menadione was investigated. The method used derived from the LUCS assay (Derick et al., 2017) and is based on the fluorescence properties of Thiazole Orange (TO) in presence of DNA. In healthy cells, TO fluorescence levels remain low due to the drug efflux transport, whereas under conditions of oxidative stress, cell homeostasis is disrupted, leading to a massive entry of the dye and consequently a huge increase in fluorescence. Thus, by measuring changes in fluorescence, it is possible to assess the potential toxicity of any cellular change, like, for instance, the influence of NQO2 and conjugating enzymes on the oxidative stress generated by, and therefore on the toxicity of, menadione. The cells were initially incubated for 1 h in the presence of TO (2 μM). The menadione was then added to the culture medium and the fluorescence intensity was immediately measured as a function of time for 4 h. The fluorescence kinetic of cells treated with menadione at doses ranging from 6 to 50 µM are presented in Figures 4,5 for SH-SY5Y-NQO2 and SH-SY5Y-NQO2-UGT, respectively. From the curves recorded with SH-SY5Y-NQO2 (Figure 5), it can easily be concluded, unsurprisingly, that the toxicity of menadione is highly dose-dependent (Sidorova and Grishanova, 2004). The increase in fluorescence, i.e., the toxic effect of menadione, is delayed when its concentration was decreased. At 50 µM, based on the slope of the first 30 min of the 2 curves recorded with or without NQO2 inhibition, it can be unambiguously concluded, as previously, that menadione toxicity increases when NQO2 is active. The same result was obtained at 25 µM even if the curve became superimposed between 1 and 2 h incubation. This result is less significant at 6 and 12 μM, however, an accelerated increase in fluorescence is observed after 2 h when NQO2 is active. From 3 h the fluorescence measured on the plate is higher. Note that, as described elsewhere, at low concentration, menadione is less toxic and can become “antioxidant” (Gironde et al., 2020). To analyze the role of the association of NQO2 with UGT1A6 the same set of experiments was carried out with SH-SY5Y-NQO2-UGT1A6 cells. The corresponding results are presented in Figure 6. In this case, whatever the concentration used, the toxic effect of menadione is considerably lower when both enzymes are active whereas, when NQO2 is inhibited which in turns corresponds to the metabolism of menadione by one electron reductases, the toxicity as measured by the TO fluorescence, is higher and increases faster. When the menadione concentration is to high the detoxifying pathway is overwhelmed and the curves get closer. One can see by comparing the times to reach the maximum in fluorescence for the 2 curves, that this difference increases when the menadione concentration decreases as if the detoxifying pathway was more efficient at low concentration. This set of fluorescence experiments demonstrates as it was the case for the determination of superoxide radicals, that the association of NQO2 with UGT1A6 can allow the enzyme to regain its detoxifying properties.

### Detection of the Menadione Glucuronide

**SCHEME 2:**
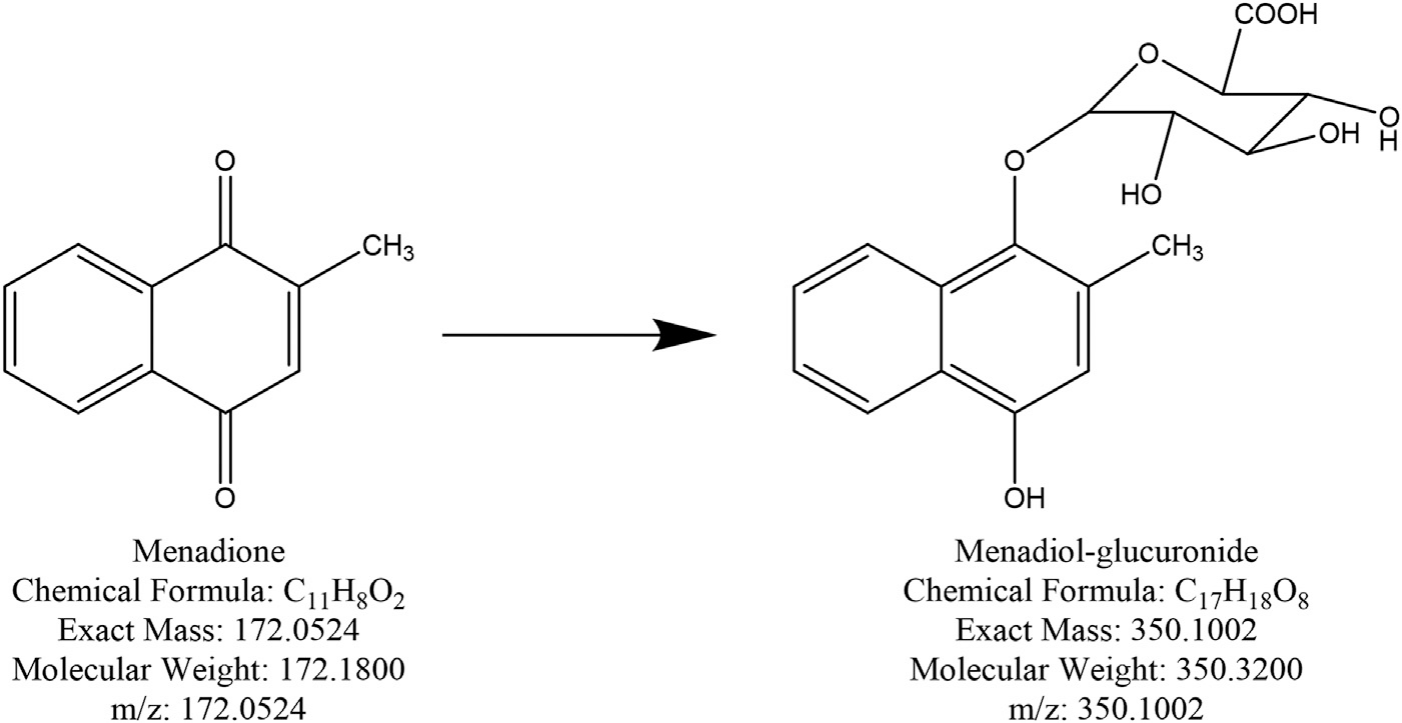
Chemical structure of menadione and menadiol-glucuronide.

**FIGURE 7:**
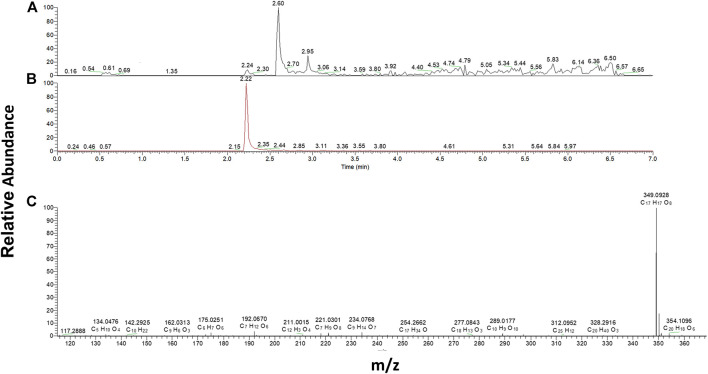
Mass-spectrometry analysis of menadiol glucuronides produced by SHY5Y5 cells. Extracted mass chromatogram of **(A)** menadione (m/z 172) and **(B)** menadiol-glucuronide (m/z 349) and **(C)** MS spectrum of menadiol-glucuronide, obtained from SH-SY5Y-NQO2-UGT1A6 homogenate after 3 h incubation with menadione (100 µM), BNAH (4 mM) and UDPGA (2 mM).

**FIGURE 8:**
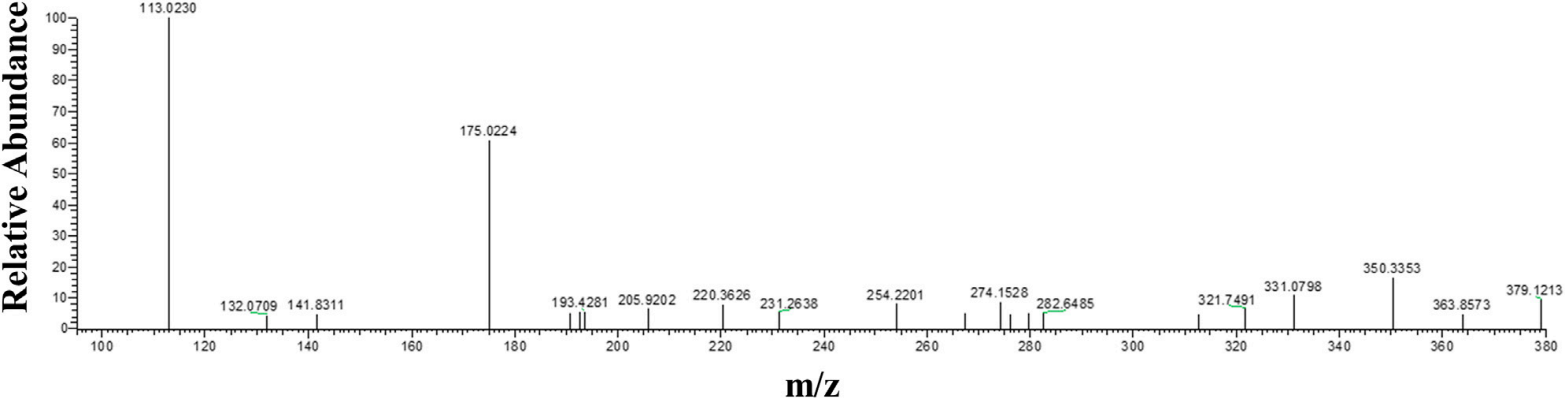
Tandem mass spectrometry analyses of menadiol glucuronides. MS/MS spectrum from parent ion m/z = 349 (retention time = 2.23 min) obtained from SH-SY5Y-NQO2-UGT1A6 homogenate after 3 h incubation with menadione (100 µM), BNAH (4 mM) and UDPGA (2 mM).

**SCHEME 3:**
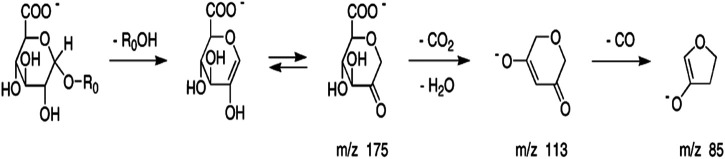
Fragmentation pathway characteristic of glucuronide.

Theoretically, when menadione is reduced to menadiol, the conjugating enzyme (here, UGT1A6) reacts with the reduced form by transferring a glucuronide group *via* an ether linkage to form the menadiol-O-glucuronide, identifiable by LC-MS through its exact mass (Scheme 2). A new set of experiments was performed on cell homogenates of SH-SY5Y-NQO2-UGT1A6 after 3 h incubation at 37°C with menadione (25 μM), BNAH (25 μM) and UDPGA (2 mM). No peak corresponding to menadiol-glucuronide was detected under these conditions, probably due to a too low amount of glucuronide produced. New tests were therefore carried out by increasing both the concentration of menadione (100 μM) but also BNAH (4 mM) since menadione is continuously recycled, leading to the concomitant consumption of the NQO2 co-substrate, BNAH. The LC-MS results are presented in Figure 7 for menadiol-glucuronide (m/z 349) and menadione (m/z 172) which is still present in the medium. In each case, the chromatogram shows a single peak at retention times: 2.60 min for menadione and 2.22 min for menadiol-glucuronide. The m/z 349 conjugated form corresponds to menadiol (174) with the addition of the glucuronide value [(176)—1 (negative ionization mode)] (C_17_H_17_O_8_). To confirm this result, LC-MS/MS tandem analyses were performed. The fragmentation of the ions corresponding to m/z 349 resulted in the characteristic fragments of glucuronide at m/z 175 and m/z 113 (Figure 8) as described in the literature (Scheme 3) (Levsen et al., 2005). In the absence of menadione, BNAH or UDPGA, no peak corresponding to menadiol-glucuronide was observed (Supplementary Figures S1–S3).

## Concluding Remarks

In the present paper, we demonstrate that using a specific neuroblastoma cells co-expressing NQO2 and the conjugating enzyme UGT1A6 together with original analytical methods, that the association of these two enzymes permit NQO2 to perform its detoxifying theoretical duty towards menadione. The conjugating enzyme acts by blocking the unstable menadiol avoiding its reoxidation and the subsequent production of ROS. This result was further confirmed by the detection of the glucuronide form of menadiol. In a more general context, after having pointed out the ambiguous role of NQO2 as a detoxifying enzyme (Boutin et al., 2019; Janda et al., 2020), we present data clearly showing that in more relevant conditions, for example in the presence of the conjugating enzyme(s), NQO2 would recuperate its detoxifying role, in line with the NQO1 one. In this pathway, NQO2 acts as a drug metabolizing Phase I enzyme, leading to an ‘activated’ molecule that can be conjugated by Phase II enzymes of the next step of drug metabolizing machinery. Interestingly, this increase the suspicion that NQO2 might have an activating role towards some quinones, particularly o-quinones, whenever this metabolism occurs in tissues/organs where the conjugation machinery is low or absent.

## Data Availability

The raw data supporting the conclusion of this article will be made available by the authors, without undue reservation.
