# Supplementary material for: Association of NQO2 With UDP-Glucuronosyltransferases Reduces Menadione Toxicity in Neuroblastoma Cells
Source: Front Pharmacol. 2021 May 10;12:660641. doi: 10.3389/fphar.2021.660641 (PMC8142080; doi:10.3389/fphar.2021.660641)
Supplement: Supplementary file 1 [file DataSheet1.docx]

**Association of NQO2 with UDP-glucuronosyltransferases reduces menadione toxicity in neuroblastoma cells.**

Monivan Chhour^a^, Pierre Perio^a^, Regis Gayon^b^, Hélène Ternet-Fontebasso^a^, Gilles Ferry^c^, Françoise Nepveu^a^, Jean A. Boutin^a,c*^, Jan Sudor^a†^, Karine Reybier^a†^

^a^ Pharma-Dev UMR 152, Université de Toulouse, IRD, UPS, 31000 Toulouse, France

^b^ Flash Therapeutics, Parc Technologique du Canal, 31400 Toulouse, France

^c^ Biotechnologie, Pharmacologie moléculaire et cellulaire, Institut de Recherches Servier, 78290 Croissy sur Seine, France

**Supplementary Data**

B

C

A

**Figure S1.** Negative control 1: Extracted mass chromatogram of (A) menadione (m/z 172) and (B) menadiol-glucuronide (m/z 349) and menadiol-MS spectrum (C) obtained from SH-SY5Y-NQO2-UGT1A6 homogenate after 3h incubation with BNAH (4 mM) and UDPGA (2 mM) without menadione.

B

C

A

**Figure S2.** Negative control 2: Extracted mass chromatogram of (A) menadione (m/z 172) and (B) menadiol-glucuronide (m/z 349) and menadiol-MS spectrum (C) obtained from SH-SY5Y-NQO2-UGT1A6 homogenate after 3h incubation with menadione (100 µM) and BNAH (4 mM) without UDPGA.

B

C

A

**Figure S3.** Negative control 3: Extracted mass chromatogram of (A) menadione (m/z 172) and (B) menadiol-glucuronide (m/z 349) and menadiol-MS spectrum (C) obtained from SH-SY5Y-NQO2-UGT1A6 homogenate after 3h incubation with menadione (100 µM) and UDPGA (2 mM) without BNAH.
